# Cross-cultural Study of the Personality Inventory for the DSM-5 (PID-5) across the Portuguese and the United Arab Emirates (UAE) Community and Clinical Populations

**DOI:** 10.2174/17450179-v18-e2207130

**Published:** 2022-08-26

**Authors:** Olga Coelho, Rute Pires, Ana Sousa Ferreira, Bruno Gonçalves, Samia A. Alkhoori, Mohamed Sayed, Amany ElRasheed, Maryam AlJassmi, Joana Henriques-Calado, Joana Stocker

**Affiliations:** 1 CICPSI, Faculdade de Psicologia, Universidade de Lisboa, Alameda da Universidade, 1649-013 Lisboa, Portugal; 2 Faculdade de Psicologia, Universidade de Lisboa, Alameda da Universidade, 1649-013 Lisboa, Portugal; 3 Instituto Universitário de Lisboa (ISCTE-IUL), Business Research Unit, Av. das Forças Armadas 1649-026 Lisboa, Portugal; 4 Rashid Hospital, Oud Metha Road, P.O. Box 4545, Dubai, UAE; 5 National Rehabilitation Center, Shakhbout City, Abu Dhabi, UAE; 6 Al Amal Psychiatric Hospital, Al Awirs Road, P.O. Box 63932, Dubai, UAE; 7 College of Natural and Health Sciences, Zayed University, P.O. Box 19282 Dubai, U.A.E; 8 Colégio de Nossa Senhora do Rosário, Avenida da Boavista, 2856,4100-120, Porto, Portugal

**Keywords:** Cross-cultural personality study, Alternative model of personality disorders, Personality traits, Arabic version of the PID-5, Portuguese version of the PID-5, Score levels

## Abstract

**Aims:**

The present paper focused on compare the PID-5 mean score levels across two matched community and clinical samples of Portugal and the UAE.

**Background:**

The generalizability and universality of the Alternative Model of Personality Disorders has been thoroughly studied through the Personality Inventory for DSM-5 (PID-5) across countries and languages. However, studies comparing Western and Middle Eastern countries are still limited, in particular those who assess the PID-5 measurement invariance.

**Objectives:**

We examined measurement invariance of the PID-5 scales across matched Emirati and Portuguese clinical and nonclinical groups, as well as compare and contrast the PID-5 mean score levels across both countries and samples.

**Methods:**

The Arabic and the Portuguese versions of the PID-5 was administered to Emirati community participants (*N* = 300, 80% women and 20% men, *M_age_* = 27.95) which were matched with Portuguese community participants (*N* = 300, 80.3% women and 19.7% men, *M_age_* = 28.96), as well as clinical participants of the UAE (*N* = 150, 61.3% women and 38.7% men, *M_age_* = 31.29) and Portugal (*N* = 150, 52% men and 48% women, *M_age_* = 44.97). We examined measurement invariance through an unrestricted Factor Analysis based program, and mean scores levels were compared and analyzed.

**Results:**

Our findings supported the PID-5 measurement invariance across the Emirati and Portuguese clinical samples pointing to the universality and generalizability of the Alternative Model of Personality Disorders. The Emirati psychiatric sample exhibited somehow higher results than the Portuguese psychiatric participants, albeit the small effect size for most of the PID-5 scales.

**Conclusion:**

Further research is needed to examine the applicability of the PID-5 across non-clinical representative samples of Portugal and the UAE, and other Middle Eastern countries.

## 1 INTRODUCTION

The degree to which culture influences the assessment, diagnosis, and treatment of Personality Disorders (PDs) is yet to be unveiled. Each culture's ethos, history and dimensions create unique contingencies that help shape the expressions of self and interpersonal functioning, as well as the promotion or suppression of specific behaviors and personality traits [1]. Consequently, what generates and perpetuates pathological patterns of personality is deeply intertwined in social conventions of emotional expression and regulation, along with behavior norms and personality traits, that go beyond the salient obvious cross-cultural differences but are often found in more subtle and subjective cultural idiosyncrasies [2, 3].

PDs are not a specific mental illness of Modern Western Societies, instead they are a severe psychiatric condition deemed to affect 7.8% of the world population [4] and in Western clinical settings, prevalence rates range from 45% to 51% in the US and 40% to 92% in European psychiatric outpatients [5]. As for the United Arab Emirates (UAE), data from primary healthcare services points to a global prevalence of 12.7% [6], contrasting with 52.7% found in Portugal [7]. However, despite the considerable variations in the prevalence of PDs worldwide, and albeit that some types are rare or even absent in certain cultures [8, 9], they all share a common denominator: the early onset, problematic diagnostic, and treatment resistance [10-12]. In fact, these differences, do not necessarily represent real cross-cultural differences, but instead mirror the difficulties in developing international guidelines and assessment tools that are scientifically valid and clinically useful to establish what can influence personality functioning and characterize PDs, globally [2, 13].

Traditional Islamic and patriarchal societies, such as the UAE, where religion overrides an act of faith but is embedded in every aspect of the individual’s life [14, 15], raise additional challenges to the diagnosis and treatment of PDs, not often answered by Western diagnostic and healing models. In the Emirati society, seeking mental health treatment is mostly a family decision that often collides with the guilt and stigma associated with a lack of faith, as a cause of mental distress. Moreover, psychological discomfort is largely expressed through metaphoric expression and communicated using oral vernacular forms of Modern Standard Arabic, not always easily captured by the clinicians in primary health care settings [15, 16]. With that being said, to what extent have the efforts in developing more culturally sensitive and evidence-based PDs nosology’s carried out by the recent editions of the Diagnostic and Statistical Manual of Mental Disorders (DSM) and the International Classification of Diseases (ICD), succeed in ways that are applicable to a Middle Eastern multicultural society, with a hybrid identity (brought about by the strong Western presence in the region), but rooted in religious and family-oriented traditions?

In both classification systems, the key innovation is the conceptualization of PDs as a dyad of severity or impairment in self and interpersonal functioning, along with the presence of maladaptive personality traits that characterize the stylistic expression of PDs. This dimensional approach, led by the Alternative Model for Personality Disorders (AMPD) published in Section III (for further studies) of the DSM-5 [17], has achieved general support, to a great extent, due to the extensive research conducted with the Personality Inventory for the DSM-5 (PID-5) [18].

The PID-5 operationalizes Criterion B of the AMPD, or the presence of 25 maladaptive personality traits in which individuals differ (facets), subsequently organized within five high order domains: Negative Affectivity, Detachment, Antagonism, Disinhibition and Psychoticism. These traits seem to be pathological extremes of the basic dimensions of normal personality, as stated by the Five-Factor Model (FFM) [19, 20], supporting its universality as well as the continuum, between normality and personality dysfunction. The PID-5 is a valuable and reliable tool that has confirmed adequate psychometric properties and replicated its factor structure across countries and samples (for a review see Somma *et al.* [21] and Zimmerman *et al.* [22]).

With the present study, we were interested in testing if maladaptive traits measured by the PID-5, are culturally shaped in their intensity and expressions, across matched clinical and non-clinical groups from two distant countries and cultures, the Middle East culture of the United Arab Emirates and the Portuguese South European culture. In other words, can findings derived from the Portuguese and Arabic versions of the PID-5 be comparable and generalized to personality research and clinical practice?

Nevertheless, before cross-cultural differences can be examined and interpreted it is crucial to establish measurement invariance to ensure that the same underlying pathological personality traits, measured by the Portuguese and the Arabic version of the PID-5, are being assessed in similar ways and have the same meaning, across the two countries [23, 24]. On this note, the PID-5 literature has already demonstrated overall measurement invariance for age [25], clinical status [23], sex [26] and cross-cultural comparisons [27]. However, despite promising results, cross-cultural comparative studies with the PID-5 are still limited to European Western countries [27, 28]. To address this gap, our study intended to extend Sorrel *et al*.’s [28] cross-cultural measurement invariance study, developed with large samples of college students from several European countries, to test the PID-5 measurement invariance also in a non-Western country, and in clinical and community samples.

Therefore, to examine measurement invariance (MI) of the PID-5 facets and domains, we used the IMINCE software [29], a free unrestricted Factor Analysis-Based program that uses Exploratory Structural Equation Modelling (ESEM) methodology, which combines features of Exploratory Factor Analysis (EFA) and Confirmatory Factor Analysis (CFA), in the sense that an EFA model is submitted to an adjustment estimation of a CFA model.

Moreover, this software is suitable for responses to items on Likert scales or dichotomic items, as it performs factor analysis using polycholic or tetrachoric correlations, and measures partial, strong, and strict invariance [29].

Accordingly, our study goals were (1) to test MI of the PID-5 scales across matched Emirati and Portuguese clinical and nonclinical groups, and (2) to compare the PID-5 mean score levels across these countries and samples. Considering the previous studies on the Emirati and Portuguese populations, we expected that the clinical groups’ mean scores would be higher than non-clinical groups.

## 2 MATERIALS AND MEHTODS

### 2.1 Ethics

The present study was approved by the Research Ethics Committee of Zayed University Dubai (ref. ZU18_36a_F), Dubai Scientific Research Ethics Committee (ref. DSREC-02/2019_07), Ministry of Health and Prevention Research Ethics Committee (Ref. MOHAP/DXB-REC/No.10/MM/2019), Deontological Committee of Psychology Faculty of the University of Lisbon (ref. Acta n.2_CD_22Oct2020), Ethic Commission of the host Portuguese mental Health institutions. All participants were carefully informed about the study and gave written consent.

### 2.2 Samples

#### 2.2.1 Community Samples

The study included an Emirati and a Portuguese convenience community sample. The Emirati community sample comprised 300 volunteer Emirati college students from Zayed University Dubai and Abu Dhabi, and their acquaintances (80% women and 20% men). The participants were aged 18 to 57 years (*M* = 27.95, *SD* = 10.19) mostly single (66.7%), had completed high school (69.3%), and at the time of the assessment, 56% were students.

The Portuguese community sample was matched based on the composition of gender and age with the Emirati community sample. A total of 300 Portuguese volunteer students and their acquaintances (80.3% women and 19.7% men), aged 18 to 60 years (*M* = 28.96, *SD* = 11.12), were recruited from the Psychology Faculty of Lisbon University. The majority of the participants were single (75.3%), had completed high school (59.5%), and at the time of the assessment were students (56.7%).

#### 2.2.2 Clinical Samples

The clinical samples were convenience samples, composed of Emirati and Portuguese psychiatric patients. The Emirati clinical sample was recruited among volunteer patients that, at the time of the assessment, were receiving treatment in mental health institutions of the UAE, namely Rashid Hospital, Al Amal Psychiatric Hospital, and National Rehabilitation Centre. The selection process was conducted by the institutions’ psychiatrists or psychologists and based on clinical records and/or clinical authority. The clinicians were asked to report the patient’s diagnosis using the DSM-5 criteria, and those who met at least one DSM-5 mental disorder were included in the study. Patients who suffered from intellectual disability, schizophrenia spectrum disorder, and major and mild neurocognitive disorders were excluded from the sample. A total of 150 inpatients and outpatients were selected, 61.3% female, 38.7% male, aged 18 to 61 years (*M* = 31.29, *SD* = 8.88). Most of the clinical participants were single (49.3%), had completed high school (66%), and at the time of the assessment, 37.3% were unemployed, 28% were employed, 15.3% were housewives, 14.7% were student, and 4% were retired.

The Portuguese clinical sample comprised 150 Portuguese volunteer psychiatric patients from mental health institutions across the country, subsequently matched (based on the diagnosis), with the Emirati clinical sample. The patients were primarily men (52%), aged 18 to 68 years old (*M* = 44.97, *SD* = 11.6). The participants were predominantly single (50%), unemployed (51.7%), and at the time of the assessment 28.2% had completed high school, and 26.2% had a bachelor’s degree or more. The predominate diagnoses for both countries’ samples were substance related disorders, depressive disorders, obsessive-compulsive disorders, anxiety disorders and bipolar disorders.

### 2.3 Instruments

Personality Inventory for DSM-5 (Krueger *et al.*, 2012 [18], Arabic version by Al-Attiyah *et al*. [30])

The PID-5 is a self-report measure that operationalizes the trait system of the DSM-5 Alternative Model of Personality Disorders. It is composed of 220 items, rated on a four-point *Likert* scale, ranging from 0 (very false or often false) to 3 (very true or often true) that characterizes 25 empirically derived lower-level facets grouped into five major domains of maladaptive personality variation. Approximately, the instrument takes 40 minutes or less to complete and it is to be used in adults (18 years old or above). The PID-5 has shown robust psychometric properties worldwide, in clinical and non-clinical samples, such as replicable factor structure, internal consistency, convergent validity with other personality measures as well as a broad range of psychopathological constructs. The UAE sample was assessed through the Arabic version of the PID-5 [30] while the Portuguese sample was studied by the Portuguese version of the PID-5 [31]. Both translated versions have proven their relevance in terms of internal consistency as well as factorial validity in clinical, and non-clinical samples [30-34].

### 2.4 Data Collection

The present study comprised two Emirati samples and two Portuguese samples. The Emirati community sample was recruited through email or in-person by the researchers. The sessions were held collectively at Zayed University Dubai and Abu Dhabi. Regarding the Emirati clinical sample, the selection of the patients was performed by the mental health units ‘clinicians and the participants were invited to take part in the study at the end of the follow-up appointments. The objectives of the study were explained, and confidentiality was emphasized. As the test requires approximately 40 minutes to apply, data collection sessions were scheduled dependent on the patients’ conditions and availability. All the participants signed a written informed consent form. As for the Portuguese community and clinical samples, data was made available by the authors of the Portuguese version of the PID-5, who also mentored this research project, and details regarding data collection are published in Pires *et al*. [31, 34].

### 2.5 Data Analysis

Statistical analyses were undertaken with the IBM SPSS (v.26, SPSS Inc., Chicago, IL) and the IMINCE software [29]. In the current study descriptive statistics of the PID-5 facets and domains for both countries and samples were obtained, internal reliability was examined through *Cronbach’s* alphas and gender effect sizes were analysed. In order to explore the normality of the scales’ distributions, the following criteria were applied: *skewness*, *kurtosis*, *Kolmogorov-Smirnov Goodness-of-fit* test (*N* > 30), and *Q-Q-plots*. Mean score differences between the UAE and the Portuguese (community and clinical) samples presenting a normal distribution, were investigated by means of paired samples *t* test and *Cohen’s d*. The effect size was considered small when *d* ≤.20, medium when .20 ˂ *d* ≤ .50, large when .50 ˂ *d* ≤ 1.0, and very large when *d* > 1.0. Accordingly, the mean score differences of the scales that presented a heteroscedastic distribution were calculated by the *Wilcoxon Signed-Rank* test, for both countries and samples. The effect size was tested through *r* = *z*/√*N*, being *N* the number of pairs without ties, and the effect size was considered small when .10 ≤ *r* < .30, medium when .30 ≤ *r* < .50 and large when *r* ≥ .50.

Furthermore, we employed the IMINCE, a free unrestricted Factor Analysis based program that allows the assessment of MI in two populations, in this particular study, the Emirati and the Portuguese community and clinical samples. Although the program is more suitable for binary or *Likert* item scores, it can as well analyse sums of item scores (facets) and sets of test scores (domains). The IMINCE examines the following invariance forms: invariance of item difficulties or intercepts, factor loadings or discriminations (partial invariance), and residual variances (strict factor invariance). This is accomplished using 1) *Cohen’s d* or univariate *t* tests or *Hotelling’s T-square* and the corresponding *F* statistics, depending on the nature of the variables involved and the size of the samples. In our case, *Cohen’s d* is the most appropriate given the large size of the UAE and the Portuguese community and clinical samples. Additionally, 2) factor loadings are obtained by an Exploratory Factor Analysis method and tested by factor congruence, factor discrepancy and approximate confidence intervals. Observed congruence and discrepancy indices are compared to the critical values *α* and, congruence indices are considered statistically significant if they are smaller than *α*, while discrepancy indices are considered statistically significant if they are larger than *α*. Finally, 3) invariance of residual variances or strict factor invariance, is assessed through percentile intervals which are obtained from a Bootstrap resampling. Nonoverlapping intervals suggest that the residual variances of a certain item are not invariant over the two populations.

## 3 RESULTS

### 3.1 Descriptive Statistics and Internal Consistency

Table **1** presents the PID-5’s facets and domains mean, *SD*, and *Cronbach* alphas of the UAE and Portuguese community and clinical samples. Overall, the PID-5 presented acceptable to good (*α* ≥ .70) alpha coefficients, in all domains and most of the facets, for both countries and samples. Higher reliability coefficients were obtained in the clinical samples compared to the community samples. The lowest alpha values were found in the facet Suspiciousness for the UAE community (.35) and clinical samples (.48), as well as in the Portuguese clinical (.59) sample. Also, the facet Manipulativeness presented the lowest alpha (.37) in the Portuguese community sample along with the facets Submissiveness (.52) and Restrictive affectivity (.55). On the other hand, the highest alpha values for both UAE (.91) and Portuguese (.89) clinical samples, were observed on Depressivity, whilst Callousness and Eccentricity reported the highest alphas in the Portuguese (.83) and UAE (.90) community samples, respectively. As for the PID-5 domains, the lowest alphas were found in the domain Disinhibition for the Portuguese community sample (.81) and UAE clinical sample (.80), along with Antagonism in the UAE community sample and Negative affectivity in the Portuguese clinical sample. The highest alpha values were displayed in the domain Psychoticism for both countries and samples.

### 3.2 Gender Effect Size

In order to prevent possible gender bias, due to uneven proportions of men and women in both, community and clinical samples, *Cohen´s d* was calculated to determine gender effect sizes. Our results pointed to very small effect sizes (*d* <.20), in some facets and domains, in both, community, and clinical samples, of the Emirati and Portuguese populations. Specifically, in the domain of Disinhibition, along with the facets of Depressivity, Distractibility and Submissiveness, in the Emirati community sample. Also, the Negative affectivity domain as well as the facets of Anxiety, Callousness, Deceitfulness, Emotional lability, Grandiosity, Irresponsibility, Restricted affectivity, and Risk taking in the Portuguese community sample. Concerning the clinical samples, the same results were found (*d* <.20) in the domain Negative affectivity in conjunction with the facets, Risk taking, Separation insecurity, and Submissiveness, in the Emirati clinical sample, together with the facets Deceitfulness and Callousness, in the Portuguese clinical sample.

### 3.3 Invariance Study of the UAE and Portuguese Community Sample

Regarding Invariance of facet difficulties or intercepts (see Table **2**), *Cohen’s d* statistic identified 10 facets with large effect sizes, between the UAE and the Portuguese community populations, namely: Callousness, Cognitive and perceptive dysregulation, Deceitfulness, Grandiosity, Intimacy avoidance, Irresponsibility, Manipulativeness, Suspiciousness, Unusual beliefs and experiences, and Withdrawal. Moreover, in terms of Invariance of factor loadings or discriminations (partial invariance), significant differences were observed in the congruence coefficients of 6 of the PID-5 facets (Anhedonia, Callousness, Eccentricity, Irresponsibility, Restricted affectivity, Withdrawal), and in the discrepancy coefficients of 9 facets (Anhedonia, Callousness, Cognitive and perceptual dysregulation, Distractibility, Eccentricity, Perseveration, Restricted affectivity, Unusual beliefs and experiences, and Withdrawal) as presented in Table **3**. As for the PID-5 domains, Detachment presented significant differences in the discrepancy coefficient along with Psychoticism in the congruence and discrepancy coefficients (Table **4**). Therefore, due to these differences, the overall congruence and discrepancy indices were also compromised. Concerning strict factor invariance in the community samples, the percentile intervals of residual variances of the PID-5 facets have shown overlapping for all facets, except for Grandiosity and Suspiciousness (see Table **5**). According to Lorenzo-Seva and Ferrando [29], nonoverlapping intervals suggest that the residual variances of a particular variable are not invariant over the populations that are being compared.

In a nutshell, our results have shown that the facets of Grandiosity and Suspiciousness, did not reach strict factor invariance, and other 10 PID-5 facets (Anhedonia, Callousness, Cognitive and perceptual dysregulation, Distractibility, Eccentricity, Irresponsibility, Perseveration, Restricted Affectivity, Unusual beliefs and experiences, and Withdrawal) along with 2 domains (Detachment and Psychoticism) did not show partial invariance across the UAE and the Portuguese community samples.

### 3.4 Invariance Study of the UAE and Portuguese Clinical Samples

In respect to the Invariance of facet difficulties or intercepts in the clinical populations, according to *Cohen’s d*, no facets presented large effect sizes (Table **6**). As for Invariance of factor loadings or discrimination (partial invariance), only the facet Separation insecurity presented significant differences in the discrepancy coefficient (Tables **7** and **8**). However, the overall discrepancy index was not compromised. Likewise, concerning strict factor invariance, the percentile intervals of residual variances of the PID-5 facets, in the clinical samples, showed overlapping for all the facets, apart from Perseveration and Separation insecurity (Table **9**). Taken together, as the residual variance is invariant over the UAE and the Portuguese populations, we can consider strict invariance for all facets and domains.

### 3.5 Group Differences

Concerning the aforementioned PID-5 scales distribution, both community and clinical samples, generally leaned to non-normality, particularly in the community samples, as only Anxiety and Emotional lability presented normal distribution. As for the clinical samples, scores were more normally distributed, specifically for 11 of the PID-5 facets and 4 of the domains. Regarding facets and domains that show non-normal distribution, in the community samples, Table **10** presents the respective mean ranks score differences *Wilcoxon Signed-Rank* test, and effect size coefficient. Non-invariant facets are presented in grey. Most of the PID-5 facets and domains results were higher and statistically significant in the UAE community sample (*p* ˂.05) compared to the Portuguese community sample. Regarding the effect size, we obtained small (.10 ˂ *r* ≤ .30) to medium (.30 ˂ *r* ≤ .50) effect sizes for 16 of the 23 PID-5 facets, with the highest effect sizes displayed by Deceitfulness (.62) and Grandiosity (.64). At the domain level, except for Negative affectivity, the UAE community sample presented significant higher results (*p* ˂.01) than the Portuguese community sample, with the highest effect size (.66) being displayed by the domain Antagonism. In respect to the variables with normal distribution, no significant differences were reported for Anxiety, *t* = 1.88, *p* =.07, despite the Portuguese community sample presented higher mean scores (*M* = 1.51; *SD* = .65) than those in the UAE sample (*M* = 1.42; *SD* = .59). Conversely, Emotional lability obtained significantly higher scores in the Portuguese sample (*M* = 1.25; *SD* = .65) compared to the UAE sample (*M* = 1.13; SD = .54), *t* = 2.43, *p* =.02, albeit the small effect size.

As for the UAE and Portuguese clinical samples, Table **11** presents the 14 non-normal distributed facets and domains, along with the mean ranks score differences, *Wilcoxon Signed-Rank* test, and respective effect size coefficient. Similarly, to the community sample, also the UAE clinical sample presented higher and statistically significant results for most of the PID-5 facets and domain (*p* ˂.05), compared to the Portuguese sample. However, the effect size coefficients obtained were small (.10 ˂ ***r*** ≤ .30) for the most part of the facets and domains. The exceptions were the facets of Deceitfulness, Grandiosity, Irresponsibility, and the Antagonism domain, with medium effect sizes (.30 ˂ ***r*** ≤.50). As for the variables with normal distribution in the clinical samples, as displayed in Table **12**, only the facets Distractibility, Hostility, Perseveration, Submissiveness and Withdrawal (*p* ˂.05), along with the Disinhibition and Psychoticism domains (*p* ˂.01) shown significant higher results in the UAE clinical sample compared to the Portuguese, though with small effect sizes (*d* ≤ .20). As expected, the mean score values of the Emirati and the Portuguese clinical groups were higher than the community groups with medium (20 < *d* ≤ .50) to very high effect sizes (*d* > 1.0).

## 4 DISCUSSION

The present manuscript addressed the applicability of the PID-5 for group comparisons across the Emirati and the Portuguese clinical and community populations. To draw valid inferences regarding the mean score differences across both countries, an MI study was previously conducted, which is a prerequisite for cross-cultural comparisons [35, 36].

Broadly, our findings have shown that all the PID-5 facets and domains can be properly compared across the Emirati and Portuguese clinical participants, but not entirely for the community participants, as just 15 of the 25 PID-5 facets, and 3 of the 5 domains proved partial invariance, from which only 12 facets have proved strict invariance. Thus, considerable caution is needed in drawing conclusions from mean trait differences on the PID-5 non-invariant scales, as they may reflect potential cultural bias [37-39]. More precisely, perhaps the two cultural/ethnic groups may be attributing different meanings to the same set of items that comprise each facet or domain [40, 41]. Another possible explanation for these differences could be that the general population has relatively low scores of pathological personality traits compared to the psychiatric population, which could prevent some dimensions of pathology from emerging in data from non-clinical groups [42, 43]. The PID-5 is by nature a clinical measure [44] comprised of items such as: “Sometimes I think someone else is removing thoughts from my head.” (Item 192), “Sometimes I feel “controlled” by thoughts that belong to someone else.” (Item 154), or “Sometimes I can influence other people just by sending my thoughts to them.” (Item 150), that reflect the extreme end of personality dysfunction, so for that, it is more likely that clusters of maladaptive trait features emerge in the psychiatric populations than among those who do not suffer from a mental disorder [42, 45].

Furthermore, it is quite common that the measurement error differs across clinical and non-clinical samples, particularly in scales assessing pathology, which impacts reliability coefficients in non-clinical groups [23], due to the presence of a limited number of individuals with high scores on maladaptive traits in the general population. In fact, although our results confirmed acceptable to good reliability coefficients across the Emirati and Portuguese clinical and non-clinical groups, for all the PID-5 domains and most of the facets, as previously reported [46, 22], the alphas of the community samples were lower than clinical samples, in both countries.

In this context, notwithstanding the importance of establishing MI, there is still little consensus on what minimum requirements would still grant practical and valid comparisons in cross-cultural personality research [47, 48]. Although ideally, strict MI should be proven to consider a measure invariant, a recent review study on personality invariance found that none of the 26 cross-cultural studies on personality assessment revised, demonstrated evidence of full scalar invariance (for a review see Dong & Dumas [49]). Thus, some authors consider it reasonable that instruments demonstrating at least partial strong invariance are suitable to establish unbiased group comparisons using personality measures [50]. Concerning our results, we highlight the need for further research to address the lack of MI in some facets and domains in the community samples, as other international studies with the PID-5 have shown no such results [23, 26, 51]. Still, we might as well consider that variation of the PID-5 structure across countries and languages could more often reflect cultural variations, instead of simply structural non-invariance [52].

Regarding the facets that showed partial invariance in the community samples, with the exception of Anxiousness and Emotional lability, all the facets presented higher mean scores in the Emirati group, though only Attention seeking, Deceitfulness, Grandiosity, Intimacy avoidance, and Manipulativeness presented medium to high effect sizes (*r* >.50). A plausible interpretation could be related with the tendency for collectivistic cultures, such as the Emirati, in adopting an acquiescent response style, which might have biased the results [53, 54]. However, considering that each society develops structures that promote different personality developments and tailor unique functional patterns [55, 56], perhaps we should only realize that the PID-5 is capturing traits of two different natures, that are not necessarily undesirable but simply mirror differences in how certain traits are cross-culturally promoted or suppressed [57]. For instance, in traditional and family-oriented societies, certain features of manipulativeness and deceitfulness, could serve as a social adaptative means to avoid direct confrontation and a diplomatic way to solve everyday social conflicts.

On the other hand, the popularity of the Western culture in the UAE, fast economic growth, and globalization, helped to create a hybrid identity, mainly among the new generations, which impacts personality functioning and consequently PDs [15]. The need for adjustment due to the acculturation process caused by the gap between the traditional and family-oriented social norms, of the Emirati society, and the European-American individualistic values and conventions [58, 59] might impose additional psychological challenges. If acculturation and multicultural co-existence, can ideally result in personal growth through a balanced integration of aspects from different cultures [1], it can also cause substantial distress when personal expectations collide with family-related expectations and social demands. Such stress has been related to anxiety disorders, substance related disorders, and increased suicide risk [60, 61]. In this context, slightly higher scores in facets related to the Antagonism domain, could mirror an adjustment process in response to new family structures, gender roles, job expectations and high educational levels, particularly among women, that have started to target leadership positions and deviate from more traditional female roles, usually linked to education or healthcare, in the Emirati society.

As for the psychiatric samples, the Emirati participants exhibited somehow higher results than the Portuguese participants, albeit the small effect size for most of the PID-5 scales. The noteworthy exceptions were Deceitfulness, Grandiosity, Irresponsibility, and the domain Antagonism, which could be mirroring relevant cultural differences, as they presented medium effect sizes (*p* ˂.01; *r* ≥ .39). If we bear in mind that the Emirati and the Portuguese samples were closely matched based on diagnosis, these results may be conceptually meaningful as they reveal cultural specificities in the intensity and expression of universal maladaptive traits across the Emirati and the Portuguese population, in clinical settings. An important outcome of our findings could be related to the stigma associated with seeking professional mental health support in Arab cultures. The fear of showing weakness or lack of faith, restrain some patients from seeking early treatment, which often happens in the late stages of mental disorders [15]. In such cases, the symptoms severity and the level of impairment are already high, which directly impacts the treatment and prognosis. Considering the dimensionality of mental disorders, and specifically of PDs, our results might be explained by differences in the level of severity of the two clinical samples.

Moreover, as expected, clinical groups´ mean scores were higher than the community groups in the Emirati and the Portuguese samples, confirming the PID-5 utility to distinguish between clinical and non-clinical individuals.

The present study has several strengths and limitations, mostly related to our sample’s composition. The major strengths were the inclusion of closely matched community and clinical samples of both countries, as well as the exclusion of non-Emirati participants, to overcome possible cultural bias, due to the high number of expats living in the UAE. On the other hand, as community and clinical samples were composed of uneven proportions of men and women, with a strong predominance of women in the community samples, *Cohen´s d* was calculated to determine gender effect sizes. Only very small effect sizes (*d* <.20) were found in some facets and domains in both countries and samples. Therefore, gender effect sizes did not seem to directly impact our results. However, our findings should be considered in light of some limitations, specifically, the young age of our participants, the high number of college students in the community samples, along with the predominance of substance related disorder diagnoses in the clinical samples. Finally, the community samples were not screened for psychopathology.

## CONCLUSION

The present study supports the PID-5 measurement invariance across the Emirati and Portuguese clinical samples pointing to the universality and generalizability of the Alternative Model of Personality Disorders trait model. Nevertheless, future research should examine the applicability of the PID-5 across representative samples of the UAE and Portugal, as well as extending our study comparisons to other Arabic and Portuguese speaking countries.

## Figures and Tables

**Table 1:** PID-5 scales’ means (*M*), standard deviation (*SD*) and Cronbach’s alphas (*α*) of the UAE and the Portuguese samples.

-	**Community Samples**						**Clinical Samples**					
	UAE			Portugal			UAE			Portugal		
**PID-5 scales**	*M*	*SD*	*α*	*M*	*SD*	*α*	*M*	*SD*	*α*	*M*	*SD*	*α*
Anhedonia	.94	.52	.77	.88	.58	.69	1.45	.60	.78	1.39	.60	.74
Anxiousness	1.42	.59	.84	1.52	.65	.81	1.84	.64	.85	1.83	.59	.62
Attention Seeking	1.11	.54	.80	.74	.61	.71	1.41	.67	.84	1.09	.74	.84
Callousness	.57	.35	.73	.30	.30	.83	.81	.52	.84	.65	.51	.83
Cognitive and Percep. Dysregul	.86	.47	.80	.51	.42	.77	1.15	.59	.86	.97	.61	.82
Deceitfulness	.86	.41	.69	.43	.41	.76	1.05	.58	.80	.75	.56	.79
Depressivity	.67	.48	.87	.61	.53	.80	1.27	.68	.91	1.10	.68	.89
Distractibility	1.04	.49	.78	.98	.62	.80	1.49	.58	.81	1.33	.64	.78
Eccentricity	.90	.57	.90	.64	.64	.81	1.27	.65	.90	1.07	.67	.87
Emotional lability	1.14	.54	.76	1.26	.64	.64	1.68	.65	.79	1.57	.68	.73
Grandiosity	1.18	.55	.71	.61	.51	.59	1.33	.65	.75	.89	.64	.72
Hostility	1.16	.49	.76	1.02	.51	.71	1.44	.71	.88	1.17	.65	.82
Impulsivity	1.00	.55	.74	.85	.63	.62	1.38	.66	.76	1.33	.69	.80
Intimacy avoidance	.89	.53	.70	.45	.55	.58	1.08	.67	.75	.98	.75	.79
Irresponsibility	.74	.42	.60	.38	.41	.71	1.21	.56	.67	.87	.64	.77
Manipulativeness	1.05	.51	.61	.68	.52	.37	1.17	.66	.75	.92	.70	.73
Perseveration	1.09	.43	.70	.89	.53	.67	1.43	.62	.84	1.27	.56	.76
Restricted affectivity	1.18	.47	.60	.86	.59	.55	1.31	.55	.64	1.17	.56	.67
Rigid perfectionism	1.38	.48	.76	1.18	.59	.79	1.55	.63	.85	1.44	.63	.80
Risk taking	1.18	.40	.74	1.05	.50	.74	1.36	.54	.82	1.27	.55	.81
Separation insecurity	1.06	.56	.75	.96	.62	.57	1.39	.68	.78	1.39	.61	.66
Submissiveness	1.03	.57	.68	.92	.67	.52	1.35	.68	.73	1.14	.67	.62
Suspiciousness	1.21	.39	.35	.96	.51	.63	1.43	.48	.48	1.43	.51	.59
Unusual beliefs and experiences	.94	.53	.75	.44	.49	.68	1.16	.67	.81	.92	.61	.74
Withdrawal	1.12	.50	.80	.70	.59	.78	1.36	.62	.83	1.18	.65	.85
Negative affectivity	1.21	.46	.88	1.25	.52	.82	1.63	.54	.90	1.60	.51	.83
Detachment	.98	.41	.87	.68	.47	.83	1.30	.51	.89	1.18	.50	.87
Antagonism	1.03	.38	.80	.57	.39	.82	1.18	.54	.88	.85	.57	.90
Disinhibition	.93	.38	.83	.73	.45	.81	1.36	.51	.80	1.18	.54	.88
Psychoticism	.90	.46	.93	.53	.44	.90	1.20	.58	.94	.98	.56	.93

**Table 2:** Facet difficulties: means (*M*) and *Cohen’s d’* statistic of the community samples.

-	**UAE Sample**	**Portugal Sample**	-
**PID-5 facets**	*M*	*M*	*Cohen’s d*
Anhedonia	.94	.88	.08
Anxiousness	1.42	1.52	-.12
Attention Seeking	1.11	.74	.46
Callousness	.57	.30	.57
Cognitive and perc. dysregulation	.86	.51	.57
Deceitfulness	.86	.43	.77
Depressivity	.67	.61	.09
Distractibility	1.04	.98	.09
Eccentricity	.90	.64	.31
Emotional lability	1.14	1.26	-.15
Grandiosity	1.18	.61	.80
Hostility	1.16	1.02	.21
Impulsivity	1.00	.85	.18
Intimacy avoidance	.89	.45	.58
Irresponsibility	.74	.38	.60
Manipulativeness	1.05	.68	.51
Perseveration	1.09	.89	.31
Restricted affectivity	1.18	.86	.42
Rigid perfectionism	1.38	1.18	.27
Risk taking	1.18	1.05	.21
Separation insecurity	1.06	.96	.11
Submissiveness	1.03	.92	.13
Suspiciousness	1.21	.96	.55
Unusual beliefs and experiences	.94	.44	.69
Withdrawal	1.12	.70	.55

Small effect *d *≤.20, medium effect size.20 < *d* ≤ .50, large .50 < *d* ≤ 1.0, and very large *d* > 1.0.

**Table 3:** Overall fit congruence and discrepancy indices per PID-5 facets in the community samples.

-	**Congruence Values**		**Discrepancy Values**	
**PID-5 facets**	Observed	Critical value (*α* =.05)	Observed	Critical value (*α *=.05)
Anhedonia	.92**	.96	.03**	.02
Anxiousness	.98	.96	.01	.02
Attention Seeking	.99	.96	.20	.03
Callousness	.79**	.86	.02**	.02
Cognitive and Percep. Dysregul.	.92	.92	.03**	.02
Deceitfulness	.94	.90	.01	.02
Depressivity	.98	.95	.01	.02
Distractibility	.97	.95	.03**	.03
Eccentricity	.85**	.94	.07**	.03
Emotional lability	.98	.96	.02	.03
Grandiosity	.98	.90	.01	.03
Hostility	.89	.87	.03	.04
Impulsivity	.96	.91	.02	.03
Intimacy avoidance	.96	.83	.01	.04
Irresponsibility	.92**	.93	.02	.02
Manipulativeness	.98	.93	.01	.02
Perseveration	.96	.95	.02**	.02
Restricted affectivity	.93**	.96	.04**	.02
Rigid perfectionism	.98	.95	.01	.03
Risk taking	.88	.87	.03	.04
Separation insecurity	.98	.90	.01	.03
Submissiveness	.95	.93	.03	.05
Suspiciousness	.91	.88	.02	.02
Unusual beliefs and experiences	.95	.80	.03**	.03
Withdrawal	.96**	.97	.03**	.02

**Significant differences

**Table 4:** Overall fit indices per PID-5 domains in the community sample.

**Domains**	**Observed Congruence**	**Critical Value** **(*α *=.05)**	**Observed Discrepancy**	**Critical Value** **(*α* =.05)**	
Negative affect.	.96	.90	.09	.10	
Detachment	.90	.88	.14**	.11	
Antagonism	.98	.87	.08	.10	
Disinhibition	.89	.87	.11	.11	
Psychoticism	.85**	.86	.13**	.11	
Overall Fit Index	.93**	.95	.55**	.40	
**Significant differences				

**Table 5:** Bias-corrected percentile intervals of residual variances per PID-5 facets in the community samples.

**PID-5 Facets**	**UAE Sample**	**Portugal Sample**
Anhedonia	(.06; .11)	(.09; .15)
Anxiousness	(.10; .17)	(.10; .18)
Attention Seeking	(.11; .17)	(.07; .15)
Callousness	(.05; .08)	(.03; .05)
Cognitive and perc. dysregulation	(.05; .08)	(.04; .08)
Deceitfulness	(.06; .10)	(.05; .09)
Depressivity	(.05; .09)	(.05; .08)
Distractibility	(.10; .14)	(.10; .16)
Eccentricity	(.06; .14)	(.12; .22)
Emotional lability	(.08; .14)	(.11; .20)
Grandiosity*	(.17; .25)	(.10; .16)
Hostility	(.09; .12)	(.08; .15)
Impulsivity	(.14; .21)	(.17; .26)
Intimacy avoidance	(.15; .22)	(.16; .25)
Irresponsibility	(.06; .10)	(.05; .08)
Manipulativeness	(.10; .15)	(.08; .13)
Perseveration	(.07; .11)	(.08; .12)
Restricted affectivity	(.09; .14)	(.08; .16)
Rigid perfectionism	(.07; .12)	(.10; .19)
Risk taking	(.09; .13)	(.11; .18)
Separation insecurity	(.14; .20)	(.16; .26)
Submissiveness	(.18; .26)	(.15; .33)
Suspiciousness*	(.08; 12)	(.13; .19)
Unusual beliefs and experiences	(.10; .16)	(.10; 20)
Withdrawal	(.08; .14)	(.07; .12)

* No overlap.

**Table 6:** Facet difficulties: means (*M*) and *Cohen’s d’ *statistic in the clinical samples.

**PID-5 Facets**	**UAE Sample** ***M***	**Portugal Sample** ***M***	***Cohen’s d***
Anhedonia	1.45	1.39	.08
Anxiousness	1.84	1.83	.01
Attention Seeking	1.41	1.09	.32
Callousness	.81	.65	.22
Cognitive and perc. dysregulation	1.15	.97	.22
Deceitfulness	1.05	.75	.41
Depressivity	1.27	1.10	.17
Distractibility	1.49	1.33	.17
Eccentricity	1.27	1.07	.22
Emotional lability	1.68	1.57	.13
Grandiosity	1.33	.89	.48
Hostility	1.44	1.17	.28
Impulsivity	1.38	1.33	.05
Intimacy avoidance	1.08	.98	.08
Irresponsibility	1.21	.87	.43
Manipulativeness	1.17	.92	.29
Perseveration	1.43	1.27	.19
Restricted affectivity	1.31	1.17	.18
Rigid perfectionism	1.55	1.44	.13
Risk taking	1.36	1.27	.08
Separation insecurity	1.39	1.39	.01
Submissiveness	1.35	1.14	.23
Suspiciousness	1.43	1.43	.00
Unusual beliefs and experiences	1.16	.92	.29
Withdrawal	1.36	1.18	.20

Small effect *d *≤.20, medium effect size .20 < *d* ≤ .50, large .50 < *d* ≤ 1.0, and very large *d* > 1.0.

**Table 7:** Overall fit congruence and discrepancy indices per PID-5 facets in the clinical samples.

	**Congruence Values**		**Discrepancy Values**	
**PID-5 Facets**	**Observed**	**Critical values (*α* =.05)**	**Observed**	**Critical values (*α* =.05)**
Anhedonia	.97	.89	.02	.06
Anxiousness	.97	.89	.01	.07
Attention Seeking	.97	.93	.03	.06
Callousness	.87	.70	.05	.13
Cognitive and perc. dysregulation	.92	.84	.04	.10
Deceitfulness	.97	.88	.02	.07
Depressivity	.96	.95	.03	.04
Distractibility	.99	.89	.01	.06
Eccentricity	.92	.87	.05	.07
Emotional lability	.96	.79	.02	.11
Grandiosity	.99	.87	.01	.08
Hostility	.93	.83	.04	.11
Impulsivity	.88	.77	.08	.15
Intimacy avoidance	.95	.66	.06	.13
Irresponsibility	.91	.86	.04	.08
Manipulativeness	.96	.92	.03	.06
Perseveration	.87	.84	.06	.08
Restricted affectivity	.94	.75	.02	.07
Rigid perfectionism	.97	.77	.02	.12
Risk taking	.90	.72	.03	.09
Separation insecurity	.85	.72	.12**	.08
Submissiveness	.85	.72	.06	.13
Suspiciousness	.78	.66	.05	.07
Unusual beliefs and experiences	.91	.72	.05	.11
Withdrawal	.98	.88	.01	.08

**Significant differences

**Table 8:** Overall fit indices per domain of the PID-5 in the clinical samples.

**Domains**	**Observed Congruence**	**Critical values (*α* =.05)**	**Observed Discrepancy**	**Critical values (*α* =.05)**	
Negative affect.	.96	.69	.13	.38	
Detachment	.96	.67	.16	.38	
Antagonism	.93	.62	.15	.40	
Disinhibition	.85	.64	.26	.38	
Psychoticism	.81	.64	.27	.38	
Overall Fit Index	.92	.90	.97	1.27	

**Significant differences

**Table 9:** Bias-corrected percentile intervals of residual variances per PID-5 facets in the clinical samples.

**PID-5 Facets**	**UAE Sample**	**Portugal Sample**
Anhedonia	(.10; .18)	(.05; .14)
Anxiousness	(.15; .25)	(.05; .13)
Attention Seeking	(.11; .19)	(.07; .16)
Callousness	(.08; .13)	(.05; .11)
Cognitive and perceptual dysregulation	(.05; .09)	(.05; .11)
Deceitfulness	(.09; .16)	(.03; .08)
Depressivity	(.07; .17)	(.06; .13)
Distractibility	(.08; .13)	(.08; .15)
Eccentricity	(.09; .16)	(.10; .20)
Emotional lability	(.14; .20)	(.12; .24)
Grandiosity	(.16; .24)	(.06; .17)
Hostility	(.16; .25)	(.10; .18)
Impulsivity	(.18; .26)	(.11; .26)
Intimacy avoidance	(.07; .21)	(.13; .45)
Irresponsibility	(.07; .15)	(.11; .19)
Manipulativeness	(.03; .18)	(.08; .15)
Perseveration*	(.11; .20)	(.06; .10)
Restricted affectivity	(.11; .17)	(.11; .19)
Rigid perfectionism	(.10; .19)	(.08; .25)
* Risk taking	(.14; .21)	(.09; .20)
Separation insecurity*	(.07; .09)	(.14; .30)
Submissiveness	(.16; .32)	(.16; .30)
Suspiciousness	(.07; .12)	(.11; .21)
Unusual beliefs and experiences	(.13; .21)	(.10; .21)
Withdrawal	(.11; .20)	(.10; .21)

*No overlap.

**Table 10:** Wilcoxon Signed Ranks Test of the PID-5 Scales in the Community Samples.

**PID-5 Scales**	**Portugal *vs*. UAE**					
	Ranks	*N*	Mean Rank	*Z*	*p*	*r*
Anhedonia	Neg.	163	137.51	-1.80	.07	.10
	Pos.	119	146.97			
Attention seeking	Neg.	198	153.42	-7.19	.00**	.41
	Pos.	87	119.29			
Callousness	Neg.	216	150.78	-9.21	.00**	.53
	Pos.	66	111.14			
Cognitive and perceptual dysregulation	Neg.	215	156.52	-9.08	.00**	.52
	Pos.	73	109.11			
Deceitfulness	Neg.	232	152.08	-10.71	.00**	.62
	Pos.	53	103.24			
Depressivity	Neg	167	139.42	1.98	.05*	.11
	Pos.	119	149.22			
Distractibility	Neg.	151	154.15	-1.75	.08	.10
	Pos.	137	133.86			
Eccentricity	Neg.	192	151.01	-5.14	.00**	.30
	Pos.	101	139.38			
Grandiosity	Neg.	226	151.24	-11.04	.00**	.64
	Pos.	52	88.46			
Hostility	Neg.	173	141.61	-3.69	.00**	.21
	Pos.	106	137.37			
Impulsivity	Neg.	168	143.2	-3.00	.00**	.17
	Pos.	114	138.99			
Intimacy avoidance	Neg.	211	149.15	-9.02	.00**	.52
	Pos.	67	109.1			
Irresponsibility	Neg.	213	142.58	-8.96	.00**	.52
	Pos.	60	117.2			
Manipulativeness	Neg.	204	142.68	-7.85	.00**	.45
	Pos.	70	122.4			
Perseveration	Neg.	186	149.19	-5.43	.00**	.31
	Pos.	98	129.81			
Restricted affectivity	Neg.	189	149.42	-.6.47	.00**	.37
	Pos.	90	120.21			
Rigid perfectionism	Neg.	179	155.01	-5.04	.00**	.29
	Pos.	108	125.75			
Risk taking	Neg.	166	154.05	-3.49	.00**	.20
	Pos.	121	130.21			
Separation insecurity	Neg.	160	145.62	-2.10	.04*	.12
	Pos.	125	139.64			
Submissiveness	Neg.	151	137.73	-2.43	.02*	.14
	Pos.	115	127.95			
Suspiciousness	Neg.	184	155.6	-6.35	.00**	.37
	Pos.	98	115.02			
Unusual beliefs and experiences	Neg.	230	154.56	-10.43	.00**	.60
	Pos.	58	104.62			
Withdrawal	Neg.	217	153.73	-8.44	.00**	.49
	Pos.	74	123.33			
Negative Affectivity	Neg.	150	143.38	-0.71	.48	.00
	Pos.	150	157.62			
Detachment	Neg.	209	166.01	-8.06	.00**	.47
	Pos.	91	114.87			
Antagonism	Neg.	244	162.24	-11.51	.00**	.66
	Pos.	55	94.59			
Disinhibition	Neg.	193	159.64	-5.64	.00**	.33
	Pos.	106	131.91			
Psychoticism	Neg.	217	167.99	-9.23	.00**	.53
	Pos.	83	104.78			

Non-Invariance facets/domains in grey. Neg. = Negative Ranks (Portuguese results < UAE results); Pos. = Positive Ranks (Portuguese results > UAE results); *p ˂.05; **p ˂.01. Small effect.10 ˂ *r* ≤ .30; Medium effect size .30 ˂ *r* ≤ .50; High effect* r* > .50.

**Table 11:** Wilcoxon Signed Ranks Test of the PID-5 scales in the clinical samples.

**PID-5 Scales**	**Portugal *vs* UAE**					
	Ranks	*N*	Mean Rank	*Z*	*p*	*r*
Anxiousness	Neg.	73	69.93	-0.35	.72	.03
	Pos.	67	71.12			
Attention seeking	Neg.	91	77.23	-3.61	.00^**^	.29
	Pos.	53	64.39			
Callousness	Neg.	84	73.38	-2.56	.01^*^	.21
	Pos.	56	66.18			
Cognitive and perceptual dysregulation	Neg.	84	79.35	-2.71	.01^**^	.22
	Pos.	61	64.26			
Deceitfulness	Neg.	89	76.83	-4.74	.00^**^	.39
	Pos.	47	52.73			
Depressivity	Neg	82	80.36	-2.23	.03^*^	.18
	Pos.	65	65.98			
Eccentricity	Neg.	88	78.35	-2.65	.01^**^	.22
	Pos.	60	68.36			
Grandiosity	Neg.	98	75.57	-5.35	.00**	.44
	Pos.	41	56.7			
Intimacy avoidance	Neg.	76	76.82	-1.08	.28	.09
	Pos.	69	68.8			
Irresponsibility	Neg.	96	72.95	-5.31	.00**	.43
	Pos.	39	55.81			
Manipulativeness	Neg.	89	72.2	-3.48	.00**	.28
	Pos.	49	64.59			
Restricted affectivity	Neg.	90	73.66	-2.14	.03*	.17
	Pos.	58	75.8			
Risk taking	Neg.	81	74.29	-1.59	.11	.13
	Pos.	63	70.2			
Unusual beliefs and experiences	Neg.	84	78.38	-3.25	.00**	.27
	Pos.	57	60.12			
Antagonism	Neg.	102	81.57	-5.18	.00**	.42
	Pos.	47	60.74			

Neg. = Negative Ranks (Portuguese results < UAE results); Pos. = Positive Ranks (Portuguese results > UAE results); *p ˂.05; **p ˂.01. Small effect .10 ˂ *r* ≤ .30; Medium effect size .30 ˂ *r* ≤ .50; High effect* r* > .50.

**Table 12:** Dependent *T*-Test results of the PID-5 facets and domains with normal distribution in the clinical samples.

-	**UAE**		**Portugal**		***T-*Test**		
**PID-5 Scales**	*M*	*SD*	*M*	*SD*	*T- *Test	*p*	*d*
Anhedonia	1.45	.60	1.39	.60	.98	.33	.08
Distractibility	1.49	.58	1.33	.64	2.11	.04*	.17
Emotional Lability	1.68	.65	1.57	.68	1.46	.15	.13
Hostility	1.44	.71	1.17	.65	3.39	.00**	.28
Impulsivity	1.38	.66	1.33	.69	.65	.52	.05
Perseveration	1.43	.62	1.27	.56	2.25	.03*	.19
Rigid Perfectionism	1.55	.63	1.44	.63	1.52	.13	.13
Separation Insecurity	1.38	.68	1.39	.61	-.11	.91	.00
Submissiveness	1.35	.67	1.14	.67	2.8	.01*	.23
Suspiciousness	1.43	.48	1.43	.51	.02	.99	.00
Withdrawal	1.36	.62	1.18	.65	2.46	.02*	.20
Negative affectivity	1.63	.54	1.60	.51	.57	.57	.05
Detachment	1.30	.51	1.18	.50	1.87	.06	.15
Disinhibition	1.36	.51	1.17	.54	3.07	.00**	.24
Psychoticism	1.20	.58	.98	.56	3.30	.00**	.27

*p ˂.05, **p ˂.01. Small effect *d *≤.20, medium effect size .20 < *d* ≤ .50, large .50 < *d* ≤ 1.0, and very large *d* > 1.0.

## Data Availability

The data that support the findings of this study are available from the corresponding author [O.C.] upon reasonable request.

## LIST OF ABBREVIATIONS

AMPD: = Alternative Model for Personality Disorders
APA: = American Psychiatric Association
CFA: = Confirmatory Factor Analysis
***d***: = Cohen’s *d*
DSM-5: = Diagnostic and Statistical Manual of Mental Disorders – 5^th^ Edition
EFA: = Exploratory Factor Analysis
*F*: = Fisher Snedecor Distribution
FFM: = Five Factor Model
IBM SPP Statistics: = IBM Statistical Package for Social Sciences
**ICD-11**: = International Classification of Mental and Behavioural Disorders – 11^th^ Edition
*M*: = Media
MI: = Measurement Invariance
*N*: = Number of participants
*p*: = Value of significance
**PD**s: = Personality Disorders
PD: = Personality Disorder
PID-5: = Personality Inventory for DSM-5
*r*: = Pearson coefficient
*r_s_*: = Spearman’s rank coefficient
*T*: = univariant *t* test
*SD*: = Standard Deviation
UAE: = United Arab Emirates
*α*: = Cronbach alpha
